# Exploring Teachers’ Familiarity and Perceptions of Preference Assessments

**DOI:** 10.1007/s40617-025-01102-8

**Published:** 2025-11-18

**Authors:** David Ray Gutierrez Miranda, Hedda Meadan, Jasmine Begeske, Samira Bashiru, John Augustine

**Affiliations:** 1 University of West Georgia, 1601 Maple Street, Carrollton, GA 30117 USA; 2 University of North Carolina, Charlotte, NC USA; 3 Purdue University, 100 N. University Street, Office 5120, West Lafayette, IN 47907 USA; 4 University of Missouri, Columbus, MO USA

**Keywords:** Preference assessments, Special education, Preschool, Perceptions, Qualitative

## Abstract

Using reinforcers and student preferences may contribute to appropriate academic and behavioral outcomes for students with disabilities. Special education teachers may identify potential reinforcers and student preferences by using preference assessment procedures, which are observation-based procedures to identify and rank student preferences. However, there is limited research on special education teachers’ knowledge of preference assessments and how frequently they use preference assessments in the classroom. The purpose of this qualitative study was to explore special education preschool teachers’ understanding, usage, and perception of preference assessment procedures. Seven special education preschool teachers in Indiana participated in study interviews. Findings revealed that teachers had little to no understanding or a very broad understanding of preference assessments, and they rarely implemented preference assessments in their classroom. Teachers believed that preference assessments are a socially valid procedure for identifying reinforcers, but most teachers did not see a need to implement them often. Potential barriers for teachers’ implementation of preference assessments are discussed.

Under Section 619 of the Individuals with Disabilities Education Act (IDEA; 2004) Part B, early childhood educators are tasked with identifying and providing individualized services to young students between the ages of 3 and 5 years old. These educators may provide services or supports to address daily functioning, academics, developmental concerns, and challenging behaviors (Boulet et al., 2009). One method to support outcomes for students with disabilities is to incorporate their preferences into the instruction and interventions they receive.

For early childhood educators, identifying student preferences can be beneficial in identifying toys or activities that would motivate young students. Researchers have shown that using salient positive reinforcers can enhance academic (Adcock & Cuvo, 2009; Akmanoglu & Batu, 2004) and behavioral outcomes (Blair et al., 2010; Mauharib et al., 2020) for students with various disabilities. For example, reinforcers has been shown to aid with receptive number identification (Akmanoglu & Batu, 2004) and engaging in classroom activities and routines (Blair et al., 2010). These outcomes can be enhanced by using highly preferred stimuli as reinforcers since using highly preferred stimuli typically leads to better student responding more consistent appropriate outcomes compared to less preferred stimuli (Graff et al., 2006; Penrod et al., 2008). The identification of effective tangible or edible reinforcers may be especially important for preschoolers as praise alone may not innately function as a reinforcer (Senn et al., 2020). Additionally, preschool teachers self-reported that they often use the “following the child’s lead” strategy, where teachers engage with and teach students during a student-selected activity (D’Agostino et al., 2024). The identification of student preferences may aid preschool teachers to identify activities the student may select. Finally, a meta-analysis has shown that just incorporating student preferences can help reduce the occurrences of undesired challenging behaviors of students with developmental disabilities (Lory et al., 2020). By reducing the occurrence of challenging behaviors, students may have more access to instructional time and opportunities for learning (Nese et al., 2021; Warren et al., 2006).

One method to identify potential reinforcers and evaluate student preferences is to conduct preference assessments. Preference assessments refer to observation-based procedures that rank the student’s preferences for items and/or activities. The highest preferred items and activities are then used as consequences when students engage in targeted behaviors and confirmed as reinforcers if the target behavior increases (Kang et al., 2013). Four preference assessments commonly cited in the literature include the (a) free-operant preference assessment (Roane et al., 1998), (b) single-stimulus (Pace et al., 1985), (c) paired-stimulus (Fisher et al., 1992), and (d) multiple-stimulus-without-replacement (MSWO; DeLeon & Iwata, 1996). For descriptions of the different types of preference assessments, we refer readers to Lill et al. (2021)’s decision making model or Johnson and Graff’s (2023) book chapter on choice and preference. Though teachers may infer student preferences, Cote et al. (2007) suggested that direct and systematic procedures, such as preference assessments, may more accurately rank and identify student preferences.

Currently, there is limited research on how preference assessments are used in practice. Graff and Karsten (2012) conducted a survey study to assess how change agents (e.g., behavior analysts, direct support professionals, and teachers) identify the preferences of individuals with developmental disabilities. The researchers found that behavior analysts were more likely to know and use preference assessments, compared to non-behavior analysts, and that non-behavior analysts, including teachers, may be less likely to use or know about preference assessments. They outlined potential barriers for implementation of these systematic procedures (e.g., lack of time and training) by teachers. In the 12 years since the Graff and Karsten article, Morris et al. (2024) was the only study that evaluated preference assessment implementation. The latter referenced study surveyed behavior analysts and found that although they reported conducting preference assessments, they may do so less than once per month. Morris and colleagues (2024) suggest that this finding is due to the common practice of behavior analysts providing choices or identifying alternative reinforcers by evaluating student behaviors.

To date, only Graff and Karsten (2012) have examined the extent to which individuals who are not behavior analysts—such as teachers—are aware of and utilize preference assessments or other strategies for identifying reinforcers and student preferences. Understanding teacher’s knowledge and their strategies to identify reinforcers and student preferences is important as using more potent reinforcers may impact the outcomes for behavioral interventions (Graff et al., 2006; Penrod et al., 2008). Furthermore, the factors that may increase or decrease a teacher’s usage of preference assessments or similar strategies are unknown. By understanding current implementation and identifying factors that hinder and support implementation, behavior analysts can better understand how to support teachers, how to modify preference assessments to be more ecologically appropriate for classroom settings, and what supports to provide to teachers. This information may be especially useful for informing research aimed toward making preference assessments more feasible (Conine et al., 2021; Weaver et al., 2017) or disseminating training to teachers (Pence et al., 2012).

Thus, the purpose of this qualitative study is to explore preference assessment implementation and knowledge among special education teachers. We chose to focus on special education preschool teachers as they provide educational services to younger children with disabilities, a population that may have more difficulty with communication (Sigafoos & Pennell, 1995). This study aimed to identify how familiar special education preschool teachers are with preference assessment procedures and how frequently they report implementing preference assessment procedures. This study also sought to build on the work of Graff and Karsten (2012) by assessing the social validity of preference assessment procedures, specifically by gathering preschool teachers’ perspectives on the goals, procedures, and outcomes associated with these assessments. Results of this study may aid researchers and practitioners to better understand (a) to what extent preference assessment procedures are understood and implemented by preschool teachers and (b) factors that influence preference assessment adoption and implementation.

This qualitative study aimed at addressing the following research questions:

1. To what extent are special education preschool teachers familiar with preference assessments?
2. To what extent do special education preschool teachers implement preference assessments?
3. How do special education preschool teachers perceive the social validity of preference assessments (e.g., benefits, feasibility, and acceptability)?

## Method

### Design

We collected data through a demographic questionnaire and an online semi-structured interview. The demographic questionnaire was used to better understand the participant characteristics and to identify potential explanations for discrepant findings. The semi-structured interview was used to explore the answers to the research questions while allowing flexibility to ask follow-up questions based on participants’ responses (Adams, 2015). Data from the semi-structured interviews were analyzed using thematic analysis with open coding (Braun & Clarke, 2006; Maguire & Delahunt, 2017).

### The Researchers

Qualitative research is interpretive in nature and the interpretation of findings can partially be influenced by who conducts the analysis (Berger, 2015; Darwin Holmes, 2020). To provide context for the readers, we have provided information about who conducted the analyses. The research team consisted of three doctoral students and two mentoring professors. The data analysis was conducted by the doctoral students. Two of the doctoral students have a background in behavior analysis and experience working within school settings while the third doctoral student has a background in special education and a limited background in behavior analysis. Both mentoring professors have experience in conducting qualitative research studies. Familiarity with preference assessments varied across members of the research team, where three authors were behavior analysts familiar with preference assessments and two authors had limited familiarity with preference assessments.

### Participants

Participants were recruited in one of three ways. First, an email invitation was sent to 34 special education directors and preschool coordinators. The directors and coordinators were then asked to forward the invitation to their preschool teachers who taught at least one student identified with a disability under IDEA. Second, we posted a recruitment flyer on the investigating institution’s Special Education Instagram and Facebook accounts. Finally, we shared the recruitment flyer through email listservs that Indiana preschool teachers were likely subscribed to such as the Indiana Head Start and Early Head Start email listserv.

Twenty participants signed a consent form to participate in the study. Participants who did not initially provide a school email address were asked to provide one prior to scheduling an interview to exclude potentially fraudulent participants. A total of nine participants provided school emails and completed an interview. Of the nine participants, two participants were later excluded, as they did not meet study inclusion criteria (i.e., provided fake school emails).

All participants identified as female and held either a bachelor’s (*n* = 3) or master’s degree (*n* = 4). Five participants received their degree in education, special education, or early education or child development (*n* = 5). The other two participants received a degree in liberal arts/theater and psychology/behavior analysis. Five participants had a teaching certification, one participant had a transition to teaching permit, and one participant did not have any education-related certifications. The age range of students in participants classrooms were 2–5 (*n* = 1), 3–5 (*n* = 4) and 3–7 years old (*n* = 2). The number of students and disabilities that made up the participants’ classrooms varied. More detailed information about participants and their classrooms can be found in Tables 1 and 2, respectively.

**Table 1:** Participant characteristics

PT	Gender	Degree	Degree focus	Licensure	Teaching experience
Molly	Female	Bachelor’s	Liberal Arts Theatre	Transition to Teaching permit	0–5 years
Peyton	Female	Master’s	Education Special Education	Teaching Certification	6–10 years
Erin	Female	Master’s	Education Special Education Early Education/Child Development	Teaching Certification	11–15 years
Marie	Female	Master’s	Psychology Behavior Analysis	None	6–10 years
Sadie	Female	Bachelor’s	Education Special Education Early Education/Child Development	Teacher’s Certification	6–10 years
Annie	Female	Master’s	Special Education	Teaching Certification	Over 20 years
Belle	Female	Bachelor’s	Education	Teaching Certification	0–5 years

**Table 2:** Classroom characteristics

PT	Student age	# of Students	# of Students w/ disabilities	Student disabilities	Level of needs
Molly	3–5 years old	Over 30	11–15	^a^ ASD, ^b^ SLI, ^c^ DD	Mild, Moderate, Severe
Peyton	3–5 years old	26–30	21–25	^a^ ASD, ^b^ SLI, ^d^ VIB, ^e^ OI, ^c^ DD	Moderate
Erin	3–5 years old	1–5	1–5	^f^ ID, ^a^ ASD, ^g^ EBD, ^b^ SLI, ^e^ OI	Moderate
Marie	2–5 years old	16–20	1–5	^f^ ID, ^a^ ASD, ^h^ ADD/ADHD, ^b^ SLI, Seizure disorder	Mild, Moderate
Sadie	3–7 years old	16–20	1–5	^a^ ASD, ^h^ ADHD, ^b^ SLI, ^c^ DD	Mild
Annie	3–7 years old	6–10	6–10	^f^ ID, ^a^ ASD, ^i^ SLD, ^b^ SLI, ^d^ VIB, ^j^ HID, ^e^ OI, Other Health Impairment	Moderate, Severe
Belle	3–5 years old	16–20	16–20	^a^ ASD, ^b^ SLI, ^c^ DD	Mild, Moderate, Severe

^a^ autism spectrum disorder, ^b^ speech and language impairment, ^c^ developmental delay, ^d^ vision impairments and blindness, ^e^ orthopedic impairment, ^f^ intellectual disability, ^g^ emotional or behavioral disorder ^h^ attention deficit disorder/attention-deficit hyperactivity disorder, ^i^ specific learning disability, ^j^ hearing impairment or deafness; students’ level of need was teacher-reported based on state level of needs metric

### Procedures

#### Data Collection

After completing an online consent form, participants were directed to an online demographic questionnaire administered on Qualtrics. Participants self-reported their gender, age, education, years of teaching experience, and student characteristics (i.e., student ages, number of students, number of students with disabilities, disability categories, level of need).

**Interviews.** Online semi-structured interviews (see example interview questions in supplemental materials) were conducted via Zoom, and participants were asked about their knowledge, experiences, and perceptions of preference assessments procedures in general and specifically about the four preference assessment procedures (free-operant, single-stimulus, paired-stimulus, MSWO). The interviews were broken into two main sections: (a) familiarity and use and (b) social validity. During the familiarity and use section, participants were asked questions to probe their knowledge about preference assessments and their perceived usage of these procedures. Participants were not given any descriptions about the preference assessments and were asked to answer the question to the best of their abilities. Participants were also reminded that they could state they were unfamiliar with the procedure.

Following the familiarity and use section, participants were given verbal descriptions and watched video examples of the four different preference assessment procedures. These descriptions and videos were developed by the first author and were shared with all participants, regardless of their prior knowledge and experience. Participants were then asked questions regarding the use of these procedures, acceptability, feasibility, barriers, and preference for specific procedures.

The first author conducted all interviews, and a second researcher was present to provide support should technology issues arise. Interviews were on average 44 min long (range 31–60 min). Transcripts were created using Zoom’s online transcription program and were reviewed by members of the research team for accuracy. Interview transcriptions were used to develop a member check that included a written summary of the participant’s responses. Participants were asked to review the summary for accuracy. All participants stated that their summary was accurate, and no participants provided any additional clarifications or corrections.

#### Data Analysis

We analyzed data from the semi-structured interviews using thematic analysis with open coding as described by Maguire and Delahunt (2017). First, we independently read through the data to become familiar with the participant responses. Then, we independently highlighted or noted texts connected to the research questions, teacher’s familiarity, usage, and/or perceptions about the preference assessments. Next, we met to discuss and develop an initial codebook based on the data. We then coded each transcript individually using the initial codebook and met to discuss coding discrepancies. Codes were refined based on the discussion. This iterative process for modifying codes occurred between each transcript, as necessary. All transcripts were re-coded using the finalized coding book. After all transcripts were coded, we developed an initial list of themes by grouping similar codes together. Finally, we checked that all themes were representative of the participant quotes and codes within them, themes consisted of multiple participant responses, and themes did not overlap within one another.

## Findings

Four themes were identified from the interview data. These primary themes were (1) mixed familiarity with preference assessments, (2) limited implementation, (3) general acceptability and reported benefits of procedures, and (4) facilitators and barriers for implementing preference assessments.

### Theme 1: Mixed Familiarity with Preference Assessments

Participant familiarity with the term “preference assessment” was mixed. Three participants (Molly, Peyton, and Annie) were not familiar with the term while four participants reported having some level of familiarity with preference assessments though not necessarily through their position as a special education preschool teacher. Specifically, Marie and Belle mentioned that preference assessments were covered during their university coursework while Erin and Sadie learned about preference assessments by working at an applied behavior analysis (ABA) clinic or working with related professionals (e.g., behavior specialists and social workers). For example, Erin, a teacher with over 10 years of teaching experience, stated, “Actually one of them [preference assessment], the first time I was introduced was by a social worker who worked with one of my kids. So yeah, through practice from other professionals.”

Although four participants were familiar with the general term “preference assessments,” most participants were not familiar with specific types of preference assessment procedures. For the free-operant, Erin and Marie had previously heard the term but could not recall how to conduct the procedure. For the single-stimulus, Erin and Marie stated they were familiar with the term. Notably, Annie and Belle stated they were unfamiliar with the single-stimulus term, but, after the researchers described the procedure, they both stated that they have previously observed a school psychologist or speech language pathologist using the procedure. For the paired-stimulus, only Marie was familiar with the procedure. Finally, only Erin and Marie were familiar with the MSWO procedure but after receiving a description of the procedure, Molly also stated that they use this procedure within their classroom.

We found that participants who stated they were familiar with preference assessments were better able to state the purpose for conducting them. The three participants unfamiliar with the procedure either described a different assessment or described a procedure where they observed how students reacted to different items. The four participants familiar with preference assessments mentioned using preference to identify rewards, with three of the four participants (Erin, Marie, and Belle) mentioning the term reinforcement or reinforcer in their responses. For example, Marie, who had degrees in psychology and behavior analysis, answered:

I feel like you can use [preference assessments] to find out what is an incentive, what is a motivator, and to kind of help better direct how to encourage their skills and development. That’s how I see it. I think it works. I like the individualism of it [in] a classroom setting.

As illustrated in Marie’s response, participants who are more familiar with preference assessments may have a deeper understanding of how preference assessments may be used to individualize supports to improve student outcomes.

In terms of conducting the procedures, most participants (*n* = 5) described conducting observations of their students. Molly described observing how the student reacts in different environments while four participants (Peyton, Erin, Marie, and Sadie) discussed providing choices to students and observing their selection. Annie described a different assessment (Indiana’s ISPROUT learning assessments; Indiana Department of Education, n.d.) and Belle described filling out a checklist based on their knowledge of the student.

### Theme 2: Limited Implementation

After participants were given a written description and watched a video of each preference assessment, they were asked whether they currently implemented the procedure and how likely they would be to implement the procedures in their classroom. For all preference assessments, most participants either stated they currently did not use the procedure or only used a similar procedure. Similarly, when asked how likely they would be to use each preference assessment, most participants stated they would implement each procedure only a few times per year or as needed. Annie, who had over 20 years of experience teaching students with moderate and severe levels of need, stated,

[I use the procedure] just kind of as needed with kid behaviors. And I mean, for a lot of this, in my opinion, if you’re doing something formal, you’re doing it so that like you, don’t have to constantly do it. You’re doing it to put something in place…you’re not changing those constantly. You may need to tweak them and hold addendums and stuff like that for that but you’re not going to be doing them all the time.

Annie’s response illustrates how teachers may feel that preference assessments may be a useful tool in certain situations, but as-needed rather than a consistent schedule.

The following section describes how often each teacher reported using and being able to implement each preference assessment. Notably, when asked to rank each preference assessment in terms of feasibility, the free-operant was stated to be the most feasible followed by the paired-stimulus, while the MSWO was listed as one of the least feasible. Molly, Erin, and Annie stated they have not used the free-operant. Peyton, Marie, and Sadie stated using a similar procedure, while only Belle stated using the free-operant. In terms of feasibility, Molly stated that she did not feel it is feasible to implement, while the remaining six participants stated they would use the free-operant either when needed or during specific points in the school year.

For the single-stimulus, Erin, Marie, and Belle stated that they have not used the procedure. Molly, Peyton, and Annie stated having used a similar procedure, while only Sadie stated having used the single-stimulus. In terms of feasibility, two participants (Molly and Erin) stated they would not use or not routinely use the single-stimulus, four participants (Marie, Sadie, Annie, and Belle) stated they would use the procedure a few times per year or as needed, and Peyton stated they would use the procedure routinely. For the paired-stimulus, Marie, Sadie, and Belle stated they had not used the paired-stimulus while Molly, Peyton, Erin, and Annie described having used a similar procedure. In terms of feasibility, Molly stated they could implement the procedure daily, while the remaining participants stated they would implement the procedure as needed or only a few times per year.

For the MSWO, five participants (Peyton, Erin, Marie, Sadie, and Belle) stated that they don’t implement the MSWO. Molly discussed having used a similar procedure during free play. Annie misunderstood how to implement the MSWO but stated she currently implements the procedure. In terms of feasibility, Peyton, Erin, Marie, Sadie, and Belle stated they would implement the procedure only a few times per year or as needed, while Molly stated they would use it daily. Annie did not state how frequently they would use the procedure.

### Theme 3: General Acceptability and Reported Benefits of Procedures

When discussing each preference assessment procedure, participants were asked about the acceptability of each procedure. However, some participants referred to the benefits of the procedure rather than the acceptability of the procedure, and thus, a few participants did not have coding related to acceptability. Across all preference assessments, all participants who discussed acceptability stated that the procedure was acceptable, and no participant stated that any procedure was unacceptable to implement in the preschool classroom. Five participants stated that the free-operant was an acceptable procedure, five participants stated that the single-stimulus was an acceptable procedure, four participants stated that the paired-stimulus was an acceptable procedure, and four participants stated that the MSWO was an acceptable procedure. When discussing the acceptability of preference assessments in general, Belle stated,

I think if your goal is to identify reinforcers and they’re relatively effective. Because, like I said in our context, we use them to generate ideas, because sometimes it’s hard to think of specific things that might work. We don’t use them a lot. Just because in our program we don’t rely heavily on extrinsic reinforcers, but we do use them for certain kids.

Although Belle’s response illustrates that they may not rely heavily on preference assessments, they believe that the procedure is acceptable and can be useful when working with certain students.

While discussing benefits, general themes across multiple preference assessments were identified and broadly categorized as (a) data-related benefits, (b) behavior-based benefits, and (c) choice-based benefits. No benefit was mentioned by more than three participants within each preference assessment, but some were discussed across preference assessments. Therefore, we will provide general discussions about the benefits across all preference assessment procedures. Table 3 shows the types of benefits discussed by the teachers based on type and preference assessment.

**Table 3:** Reported benefits from implementation

Type of benefit	Benefits	Preference assessment	Participant
Data-Related Benefits	Identify Preferences & Motivation	Free-operant	Sadie, Annie
		Single-stimulus	Sadie, Annie
		Paired-stimulus	Molly, Annie, Belle
		^a^ MSWO	Molly, Peyton, Belle
	Monitor Student Preferences	Free-operant	Marie
		Single-stimulus	Marie
	Gives Useful Data	^a^ MSWO	Peyton, Marie
	Useful Follow-up Assessment	^a^ MSWO	Marie, Belle
Behavior-Based Benefits	Build Rapport w/ Student	Free-operant	Belle
		Single-stimulus	Molly, Marie
	Less Challenging Behaviors	Free-operant	Marie
	Increasing Desirable Behaviors	General/Overall	Erin, Sadie, Annie
Choice-Based Benefits	Allows for More Choice	^a^ MSWO	Marie
	Doesn’t Burden Student with Choices	Single-stimulus	Marie
		Paired-stimulus	Molly
	Allows Free Choice to Students	Free-operant	Erin, Belle

^a^ multiple-stimulus-without-replacement

For data-related benefits, participants discussed how different preference assessments could help them to identify their students’ preferences and motivations. For each preference assessment at least two participants discussed this type of benefit. Additionally, Marie stated that the free-operant and single-stimulus could help them monitor student preferences over time. The usefulness of the data can be depicted in the following quote from Annie:

I really like it. I think it’s really interesting to have it be like I don’t want to say student driven, but there may be things in there that you never thought that they would like that. They gravitate the most to, even though they have other toys that you would have thought be a bigger motivator to them. So, I like the data collection piece of that.

Annie’s response shows how preference assessments can be used to uncover useful data that may be unexpected.

For behavior-based benefits, participants primarily discussed how preference assessments could aid in relationship-building or desired changes in student behaviors. For example, Sadie described how preference assessments have been an essential tool for behavior management:

Yeah, they’re [preference assessments] integral. They’re really important for my behavior plans. One thing, when the student starts to shut down. I can be like, Okay. Well, remember, this is the things they like to do. So, bring those up and like, do your worksheet. If you want to do this, you do this [task] if you want to do this [reinforcer].

Through their response, Sadie highlighted how they have used preference assessments to motivate students to complete class tasks.

For choice-based benefits, participants discussed how the number of choices or the free selection of choices could be beneficial. Specifically, participants stated that the MSWO would allow for more choice, the single-stimulus and paired-stimulus would not burden students with too many options, and the free-operant allows students with free choice.

### Theme 4: Facilitators and Barriers for Implementing Preference Assessments

While discussing feasibility of each preference assessment, participants were asked if there were any factors that may make them more or less likely to implement each procedure. A few participants mentioned facilitators that may make them more likely to implement the preference assessment, but participants primarily discussed barriers that would limit their implementation of each preference assessment. Within each preference assessment, no single facilitator or barrier was mentioned by more than four participants, but a few were discussed across preference assessments. Thus, we will discuss general facilitators and barriers across all preference assessment procedures. A list of the facilitators and barriers discussed by the participants can be found in Tables 4 and 5, respectively.

**Table 4:** Facilitators for implementation

Type of facilitator	Facilitator	Preference assessment	Participant
Classroom-Related Facilitators	Sufficient Staffing	Free-operant	Peyton
	Sufficient Staffing	Single-stimulus	Peyton
	Environmental Arrangement	Free-operant	Marie, Belle
Student-Related Facilitators	Student Satiating on Current Reinforcer	Free-operant	Annie
	Student Has Prerequisite Skills	Paired-stimulus	Peyton
Teacher-Related Facilitators	Easy to Implement	Free-operant	Annie
	Easy to Implement	Single-stimulus	Marie
	Easy to Implement	Paired-stimulus	Belle
	Personal Preference for Procedure	Paired-stimulus	Erin
Need-Based Facilitators	Recommended by Behavior Specialist	^a^ MSWO	Annie
	Student’s Level of Challenging Behavior Increasing	Paired-stimulus	Annie
	Need to Introduce New Stimuli	Single-stimulus	Sadie

^a^ multiple-stimulus-without-replacement

**Table 5:** Barriers to implementation

Type of facilitator	Facilitator	Preference assessment	Participant
Classroom-Related Facilitators	Time/Effort to Conduct	Free-operant	Peyton
	Time/Effort to Conduct	Single-stimulus	Marie
	Time/Effort to Conduct	Paired-stimulus	Peyton, Erin, Marie, Annie
	Time/Effort to Conduct	^a^ MSWO	Erin, Marie, Belle
	Time/Effort to Conduct	Free-operant	Peyton, Erin
	Lack of Time	Single-stimulus	Molly
	Lack of Time	Paired-stimulus	Erin, Belle
	Lack of Resources/Staff	Single-stimulus	Molly
	Lack of Resources/Staff	Paired-stimulus	Molly, Erin
	Lack of Resources/Staff	^a^ MSWO	Erin
	Environmental Arrangement	Single-stimulus	Erin
	Environmental Arrangement	Paired-stimulus	Marie
Student-Related Barriers	Student Lacks Requisite Skills	Free-operant	Marie, Belle
**Type of facilitator**	**Facilitator**	**Preference assessment**	**Participant**
Student-Related Barriers	Student Lacks Requisite Skills	Paired-stimulus	Belle
	Student Lacks Requisite Skills	^a^ MSWO	Payton, Belle
	Student’s Challenging Behavior	Single-stimulus	Peyton, Erin, Annie, Belle
	Student’s Challenging Behavior	Paired-stimulus	Molly
	Student’s Challenging Behavior	^a^ MSWO	Molly
	Too Many Choices	Paired-stimulus	Peyton
	Too Many Choices	^a^ MSWO	Peyton
Teacher-Related Barriers	Personal Preference in Procedures	Single-stimulus	Marie, Sadie
	Personal Preference in Procedures	Paired-stimulus	Peyton
	Personal Preference in Procedures	^a^ MSWO	Peyton, Marie
	Data Not Useful	Free-operant	Erin
	Data Not Useful	Single-stimulus	Peyton, Belle
	Data Not Useful	Paired-stimulus	Sadie, Belle
	Data Not Useful	^a^ MSWO	Peyton, Erin, Annie

^a^ multiple-stimulus-without-replacement

#### Facilitators

A few participants discussed factors that may increase their usage of preference assessments. Most facilitators were mentioned only once by a participant; however, there were two notable facilitators. First, environmental arrangement was the only facilitator to be discussed by two participants when discussing the same preference assessments. Both Marie and Belle stated that they may be more likely to use the free-operant because the procedures align with how their classroom is already structured. For example, when explaining why they thought the free-operant was feasible to implement, Belle stated,

It’s just because it’s very integrated into the way our classroom already runs, so you wouldn’t be battling behaviors when you’re trying to do the assessment. You could really just focus on figuring out which things are interesting to them. And if you do enough observations over time, I think it would help with the limitation of sometimes they’re only going to pick one or two, and like the observation period. But if you do it a couple of times, you might be able to get a little bit—a few more options than just that.

Belle’s response highlights how the alignment between the environment and the procedure may make it easier to conduct the assessment. Specifically, Belle stated that her class engages in free choice centers where students explore the toys and activities in the different centers, and she would occasionally jot down what students were engaging with at specific centers. Belle’s current classroom structure and practices share similarities to conducting the free-operant.

Second, three participants discussed the facilitator that a procedure was easy to implement. However, all three participants had brought up this facilitator for a different preference assessment. When discussing the single-stimulus, Peyton stated,

I see it [single one] as something that you don’t even have to have this like ornate kind of plan. You literally can say this afternoon, I’m [going to] pull a couple of students aside, and you, have it, we have enough teachers in the room where I think that one’s easily, will be easily implemented.

Through this quote, Peyton highlights how the simplicity of the procedure may increase her perceived use of the procedure, even if hypothetically.

#### Barriers

Participants discussed multiple barriers to preference assessments implementation, including classroom-related barriers, student-related barriers, and teacher perceptions. For classroom-related barriers, the most discussed barrier was the time and effort needed to conduct the preference assessments and this barrier was brought up for all preference assessments. This barrier was reported more for the paired-stimulus (*n* = 4) and MSWO (*n* = 3). Relatedly, teachers reported having a lack of time, resources, or staff as barriers preventing them from implementing the procedures. Molly shared, “There has to be time set aside for me to do that with them [students], and that means that I have to have more adults in my room that will help watch my kiddos.” Molly’s response not only illustrates how these barriers constrain a teacher’s ability to implement preference assessments but also how these barriers may not occur in isolation.

Student-related barriers included students lacking prerequisite skills, students engaging in challenging behaviors (e.g., tantrums), and the risk of burdening the student with too choices. The theme of students not having prerequisite skills were discussed by Peyton, whose classroom includes students with moderate levels of support needs, and Belle, whose classroom includes students with mild, moderate, and severe levels of support needs. These two teachers noted that some of their students may struggle to select only one option or may be unable to make selections when more than two choices are presented. Additionally, multiple participants discussed difficulties related to challenging behaviors, specifically with the single-stimulus. This concern was described by Peyton, a teacher who teaches 21–25 students with disabilities who have moderate levels of support needs:

The [meltdowns] can be pretty intense…My biggest barrier about switching things if they lose interest. That’s one thing. But for me to just say, Oh, time’s up, if I haven’t given them a warning, you know I’m like, Okay, almost finished. And I show I have a stoplight card, and if I show them the yellow, almost finished, then they know that I’m gonna take it away and give them something else.

Through this response, Peyton describes how the removal of a preferred toy may evoke challenging behaviors and the effort they go through to mitigate the occurrence of challenging behaviors.

Finally, for teacher-related barriers, participants discussed preferences for other methods and doubted the usefulness of the data collected. Notably, participants were skeptical about the data’s usefulness for different preference assessments. For the free-operant, Erin stated they could identify preferences through less structured observations. Peyton also had similar sentiments when discussing the single-stimulus and stated that the data are not useful as she already knows what the student likes. Peyton stated:

I’m not always right. I don’t want to make it sound that way, but I guess I feel like I know what they’re going to pick. So, let’s just cut to the chase and just give them that preferred [item] right away. Because I’m usually trying to prevent a meltdown of some sort, or to keep them at the table to keep them sitting.

Peyton’s statement indicates that some teachers may have some beliefs that they can accurately identify student’s preferences, and as a result, they believe they do not need to conduct preference assessments. For the single-stimulus, Peyton and Belle also discussed concerns about not being able to compare and identify preferences. For the paired-stimulus, Sadie stated that they only need to identify their favorite item and did not need a hierarchy while Belle was unsure about how reliable data would be if participants were making a forced choice. For the MSWO, participants expressed confusion about what type of data and its usefulness because selected items would consistently be removed or moved around. These teacher concerns and confusion indicate that teachers may need more in-depth explanations about why procedures are conducted a specific way (e.g., rotating each item after selection to better identify positional biases).

## Discussion

Preference assessments are procedures that can reliably identify highly preferred stimuli that may act as putative reinforcers to use in behavioral interventions (Kang et al., 2013). Furthermore, researchers also suggest that preference assessments may more reliably identify putative reinforcers than teacher recommendations (Cote et al., 2007). Because special education teachers may often implement behavioral interventions, they may benefit from understanding and implementing preference assessments with their students who have disabilities. Preference assessments may also help teachers identify highly preferred stimuli to incorporate during pairing sessions or during academic tasks, which may lead to enhanced student engagement and outcomes (Jerome & Sturmey, 2014; Lory et al., 2020; Nese et al., 2021; Warren et al., 2006). Owing to the limited research on teachers’ beliefs and usage of reference assessments, the purpose of this study was to explore how special education preschool teachers understand and implement preference assessments.

In this study, we found that all special education preschool teachers were either unfamiliar with or had a broad understanding of preference assessments. Additionally, we found that teachers rarely used preference assessments in their classrooms and relied more heavily on informal procedures (e.g., observations without data collection). These findings support the findings of Graff and Karsten (2012) who found that individuals who work in schools infrequently implemented preference assessments and suggested that this low usage was potentially due to lack of training. Researchers reported that limited knowledge and training for behavioral interventions or procedures may be associated with a teacher’s use of the intervention or practice (Chezan et al., 2023; McNeil, 2019). Fortunately, there are various training procedures that can effectively teach educators to implement preference assessments with high fidelity. For example, research has evaluated the use of behavioral skills training (O’Handley et al., 2021; Weston et al., 2020), video-based training (Lipschultz et al., 2015; Nottingham et al., 2017), and computer-based instruction (Marano et al., 2020; Vladescu et al., 2022).

Teachers discussed multiple barriers that limited how often they would use preference assessments. The most frequently discussed barriers were related to lack of time and resources alongside the effort and time needed to implement the procedure. These findings echo those of Chezan et al. (2023) who found that teachers were less likely to implement behavioral interventions due to these same factors. These findings highlight the importance for researchers and behavior analysts to understand the natural contingencies and resources within the classroom when supporting teachers. The development of strategies or procedures to make preference assessments less-resource intensive or better fit the demands of classroom settings may aid in increasing teacher buy-in and usage of preference assessments. Strategies such as providing embedding choice trials throughout the day or conducting the free-operant during student breaks may make conducting preference assessments more feasible (MacNaul et al., 2021).

Additionally, teachers also expressed concerns about evoking challenging behaviors and felt confused about the necessity of specific procedures and data (e.g., need for hierarchical data). These concerns are important for researchers and behavior analysts to consider as they may indicate a teacher’s assumption and negatively affect teacher buy-in. When researchers and behavior analysts can identify that a teacher has these concerns, they may address the concern or provide an alternative solution. For example, a behavior analyst may suggest using the free-operant for students who engage in challenging behaviors or may provide an explanation for using hierarchical data (e.g., we can identify the second highest preferred item to use when the highest preferred item is unavailable).

When evaluating teacher’s perceptions about the procedures, teachers stated that all preference assessments were acceptable to use in the classroom and noted some potential benefits. Most notably, all but one teacher indicated that conducting a preference assessment would provide valuable data regarding reinforcers and student motivation. Though they believe preference assessments may be useful tools, most teachers stated they would potentially implement the procedures only as needed or a few times a year. Research on preference stability may be useful in providing initial guidance on how often preference assessments should be conducted. For example, MacNaul and colleagues (2021) conducted a review on preference stability and found that some preference assessments provide more consistent results between preference assessment administrations (e.g., paired-stimulus is more consistent than the free-operant). On the basis of MacNaul et al.’s (2021) findings, we may provide general recommendations to teachers that the paired-stimulus should be conducted at least monthly, while other preference assessments, such as the free-operant should be conducted more frequently.

## Limitations and Implications

As with many studies, the current study has some limitations that may affect its generalizability. First, our sample consists of seven Indiana special education preschool teachers. Because all participants are limited to one state and only to preschools, the external generalizability of our results may be limited. Additionally, it is important to note that data on cultural identifiers of teacher participants were unintentionally not collected, which limits the generalizability to diverse and broader populations. If more data are collected across the United States and across different grade levels, researchers and behavior analysts may better understand the barriers and perceptions that may limit teachers from using preference assessments. This broader understanding of the barriers and experiences may better inform behavior analysts on how to best adapt or modify preference assessment procedures to address teachers’ concerns. Second, while this study attempted to examine the extent to which teachers know and implement preference assessments in their classrooms, the results of this study are based on teacher self-report, which may not accurately reflect their actual knowledge and usage of preference assessments. Future research may conduct observations to verify teacher’s usage of practices. Finally, our current study only provided participants with a brief description and short video of each preference assessment. Although this allowed us to give participants a general overview of each preference assessment, participants may not have fully understood the procedures and may have responded differently than if they had high familiarity. Teacher knowledge of and confidence with behavioral interventions may be associated with their usage of the intervention (Chezan et al., 2023; McNeil, 2019); therefore, it may be important to assess teacher’s prior experiences with preference assessments and provide training, so teachers feel competent in implementing the procedure.

When providing training to teachers, teachers may not have any familiarity with these procedures, and it may be helpful to tailor training materials. For example, Graff and Karsten (2012) found that providing teachers with written descriptions that were free of jargon and providing diagrams can be effective in training teachers to implement the paired-stimulus and MSWO with high fidelity (90% accuracy over 2 consecutive sessions). Additionally, training can also incorporate video models that visually show how the whole procedure and each step is conducted (Nottingham et al., 2017). Additionally, teachers may be confused about certain procedural elements such as why preference hierarchies are important or why stimuli are shifted during the MSWO. It may be helpful for training to have time for teachers to ask questions or for training to include sections that address common concerns or confusions about implementing preference assessments. For example, when discussing identifying preferences and ranking stimuli, trainers may state that preference hierarchies allow us to quickly identify the next most preferred toy when the highest preferred toy is unavailable (e.g., toy broke, batteries died, or ran out of favorite snack). Finally, teachers may work with students of varying levels of support needs, and they may benefit from training that provides information on how to select the most appropriate preference assessment procedure based on student characteristics. Readers may refer to the most recent preference assessment decision-making models SPADs, which was developed by Lill and colleagues (2021).

Behavior analysts supporting teachers may also want to consider classroom environments and the current contingencies placed upon teachers. Common facilitators and barriers discussed in interviews were related to limited classroom resources (e.g., time and staffing) or student characteristics (e.g., student’s challenging behaviors and prerequisite skills). Behavior analysts should be mindful of teachers’ concerns and make attempts to identify the most appropriate preference assessment based on their concerns. For example, if a teacher is concerned that the student will get aggressive when preferred items are removed during the assessment, the behavior analyst may recommend that instead of using selection-based preference assessments, the teacher should use the free-operant preference assessment as the student has free access to all the presented items throughout the whole assessment. Alternatively, behavior analysts may recommend adaptations that make the preference assessment more feasible to conduct. For example, behavior analysts may recommend to a teacher with limited time and staff to (a) conduct a 6-item paired-stimulus rather than a 10-item paired-stimulus, (b) allow teachers to use longer intervals for the free-operant, or (c) embed paired-stimulus choice trials throughout the day rather than during one sitting. By considering the teacher’s concerns, behavior analysts may be more likely to gain teacher buy-in and implementation.

## Data Availability

The de-identified datasets generated during the current study are available from the corresponding author upon reasonable requests.
